# Real-life use of vitamin D_3_-fortified bread and milk during a winter season: the effects of *CYP2R1* and *GC* genes on 25-hydroxyvitamin D concentrations in Danish families, the VitmaD study

**DOI:** 10.1007/s12263-014-0413-7

**Published:** 2014-06-17

**Authors:** Janna Nissen, Ulla Vogel, Gitte Ravn-Haren, Elisabeth W. Andersen, Bjørn A. Nexø, Rikke Andersen, Heddie Mejborn, Katja H. Madsen, Lone B. Rasmussen

**Affiliations:** 1 Division of Nutrition, National Food Institute, Technical University of Denmark, Mørkhøj Bygade 19, 2860 Søborg, Denmark; 2 National Research Centre for the Working Environment, 2100 Copenhagen, Denmark; 3 Division of Toxicology and Risk Assessment, National Food Institute, Technical University of Denmark, 2860 Søborg, Denmark; 4 Department of Applied Mathematics and Computer Science, Technical University of Denmark, 2800 Lyngby, Denmark; 5 Department of Biomedicine, Aarhus University, 8000 Aarhus, Denmark

**Keywords:** SNPs, Vitamin D, 25-Hydroxyvitamin D, D_3_-fortification, Genetic risk score, Vitamin D insufficiency

## Abstract

Common genetic variants rs10741657 and rs10766197 in *CYP2R1* and rs4588 and rs842999 in *GC* and a combined genetic risk score (GRS) of these four variants influence late summer 25-hydroxyvitamin D (25(OH)D) concentrations. The objectives were to identify those who are most at risk of developing low vitamin D status during winter and to assess whether vitamin D_3_-fortified bread and milk will increase 25(OH)D concentrations in those with genetically determined low 25(OH)D concentrations at late summer. We used data from the VitmaD study. Participants were allocated to either vitamin D_3_-fortified bread and milk or non-fortified bread and milk during winter. In the fortification group, *CYP2R1* (rs10741657) and *GC* (rs4588 and rs842999) were statistically significantly associated with winter 25(OH)D concentrations and *CYP2R1* (rs10766197) was borderline significant. There was a negative linear trend between 25(OH)D concentrations and carriage of 0–8 risk alleles (*p* < 0.0001). No association was found for the control group (*p* = 0.1428). There was a significant positive linear relationship between different quintiles of total vitamin D intake and the increase in 25(OH)D concentrations among carriers of 0–2 (*p* = 0.0012), 3 (*p* = 0.0001), 4 (*p* = 0.0118) or 5 (*p* = 0.0029) risk alleles, but not among carriers of 6–8 risk alleles (*p* = 0.1051). Carriers of a high GRS were more prone to be vitamin D deficient compared to carriers of a low GRS. Furthermore, rs4588-AA carriers have a low but very stable 25(OH)D concentration, and interestingly, also low PTH level.

**Electronic supplementary material:**

The online version of this article (doi:10.1007/s12263-014-0413-7) contains supplementary material, which is available to authorized users.

## Introduction

In northern latitudes (>40°N), low vitamin D status in humans, measured as 25-hydroxyvitamin D (25(OH)D) concentrations, is common during winter months. This is because vitamin D cannot be synthesized in the skin due to the lack of solar ultraviolet B radiation (UVB) and because the average dietary intake of vitamin D is insufficient (Thuesen et al. 2012). Moreover, twin- and family-based studies indicate that genetic factors may influence 25(OH)D concentrations appreciably (Engelman et al. 2008; Shea et al. 2009; Karohl et al. 2010). Two genome-wide association studies (GWAS) and several candidate gene studies have shown single-nucleotide polymorphisms (SNPs) to influence 25(OH)D concentrations (Engelman et al. 2008; Sinotte et al. 2009; Bogh et al. 2010; Ahn et al. 2010; Bu et al. 2010; Zhang et al. 2012; Monticielo et al. 2012; Engelman et al. 2013; Zhang et al. 2013; Nissen et al. 2014). These SNPs are located in the group-specific component also known as Gc globulin (*GC*) and in or near genes involved in vitamin D synthesis, activation or degradation. These findings indicate that 25(OH)D concentrations do not only depend on vitamin D intake and sun exposure, but also on genetic factors. Thus, genetic factors may help to identify individuals at risk of low vitamin D status.

We have previously found genetic variants in *CYP2R1* (rs10741657 and rs10766197) and *GC* (rs4588 and rs842999) genes to predict late summer 25(OH)D concentrations in Danish families in a study of 25 SNPs in vitamin D metabolism (Nissen et al. 2014). The main focus of this study is therefore on the influence of rs10741657 and rs10766197 in *CYP2R1*, and rs842999 and rs4588 in *GC* on 25(OH)D concentrations in participants allocated to either vitamin D_3_-fortified bread and milk or non-fortified bread and milk during winter.

*CYP2R1*, a member of the cytochrome P450 family of enzymes, is the primary enzyme that hydroxylates vitamin D to 25(OH)D in the liver. Genetic variants of the *CYP2R1* gene are strongly associated with 25(OH)D concentration (Wjst et al. 2006; Ramos-lopez and Brück 2007; Bu et al. 2010; Zhang et al. 2012, 2013; Nissen et al. 2014) and reached a high score in two GWAS (Ahn et al. 2010; Wang et al. 2010). Furthermore, Ahn et al. (Ahn et al. 2010) observed heterogeneity between different cohorts in the GWAS and the association of 25(OH)D concentration with *CYP2R1*. A missense mutation in *CYP2R1* in exon 2 (L99P) is known to lead to vitamin D deficiency (Cheng et al. 2004).

Genetic variants in the *GC* gene reached the highest score in two GWAS (Ahn et al. 2010; Wang et al. 2010), and several candidate gene studies have found association with 25(OH)D concentrations (Lauridsen et al. 2005; Kurylowicz et al. 2006; Abbas et al. 2008; Engelman et al. 2008; Sinotte et al. 2009; Fu et al. 2009; Gozdzik et al. 2011; Lu et al. 2012; Nissen et al. 2014).

The *GC* gene encodes the vitamin D-binding protein (DBP), which is the primary vitamin D carrier protein. DBP binds with high affinity 85–90 % of circulating 25(OH)D, albumin binds with low affinity 10–15 % of circulating 25(OH)D and less than 1 % of 25(OH)D is in the free form (Bikle et al. 1986). The main function of DBP is to stabilize and prolong the half-life of 25(OH)D and other vitamin D metabolites (Speeckaert et al. 2006). DBP has several other important biological functions including fatty acid transportation, extracellular actin scavenging, leucocyte C5a-mediated chemotaxis, macrophage activation and stimulation of osteoclasts (Pekkinen et al. 2014).

The most studied *GC* SNPs are rs4588 and rs7041 that give rise to three common DBP isoforms, *GC1F* (rs7041-T, rs4588-C), *GC1S* (rs7041-G, rs4588-C) and *GC2* (rs7041-T, rs4588-A), which differ by amino acid composition and glycosylation (Gozdzik et al. 2011). Vitamin D status differed significantly depending on rs4588 (or rs2282679, *r* ^2^ > 0.99) and/or rs7041 genotypes, where the A-allele of rs4588 and/or the T-allele of rs7041 were consistently associated with lower 25(OH)D concentrations (Lauridsen et al. 2005; Kurylowicz et al. 2006; Abbas et al. 2008; Engelman et al. 2008; Sinotte et al. 2009; Fu et al. 2009; Gozdzik et al. 2011; Lu et al. 2012). In Caucasian, rs4588 and rs7041 are in almost complete linkage disequilibrium (LD) (Haploview software version 4.2). There is biological support that the affinity to both 25(OH)D and 1,25(OH)_2_D is higher for the rs4588 C-allele isoform than for the A-allele isoform (Arnaud and Constans 1993). Based on glycosylation patterns, it is suggested that the *GC2* phenotype is fast metabolizer. Kawakami et al. (1981) observed that the metabolic rate indeed was higher in *GC2*-*2* individuals than in *GC1*-*1* individuals. In addition, the *GC2* genotype, which is associated with lower 25(OH)D concentrations, is also associated with low mean DBP concentration (Lauridsen et al. 2005). The *GC2* and *GC1S* isoforms are more frequent in people with light skin whereas the GC1F isoform is more frequent in people with dark skin (Kamboh and Ferrell 1986).

Measurement of 25(OH)D concentration in blood is currently the best biological marker of vitamin D status and reflects total vitamin D exposure—from diet, supplements and cutaneous synthesis. Severe vitamin D deficiency (<12 nmol/L) is a medical condition associated with osteomalacia in adults and rickets in children. Vitamin D deficiency can lead to osteoporosis due to increased bone resorption caused by increased serum concentrations of parathyroid hormone (PTH) (Holick 2007). Moreover, vitamin D deficiency is associated with muscle weakness, falls and osteoporotic fractures (Lips and van Schoor 2011). Maintaining a sufficient vitamin D status (>50 nmol/L) is important, not only for bone health, but also because vitamin D deficiency may be associated with various non-skeletal health outcomes (Borradale and Kimlin 2009). Thus, a sufficient vitamin D status may have a disease risk-reduction potential (Grant 2011). Moreover, a U-shaped association exists between 25(OH)D concentrations and risk of cardiovascular disease, certain cancers and overall mortality (Ross et al. 2011).

There is an on-going international discussion regarding which cut-off values should define sufficient 25(OH)D concentrations. There is a general agreement that a 25(OH)D concentration of at least 50 nmol/L is sufficient (Ross et al. 2011; Nordic Council of Ministers 2014). Concurrently, some experts argue that a 25(OH)D concentration >75 nmol/L is required to achieve sufficient vitamin D status and non-skeletal benefits (Holick and Chen 2008; Zhang and Naughton 2010).

It is not easy to determine which doses of vitamin D are required to achieve sufficient 25(OH)D concentrations. The Institute of Medicine (IOM) recently reported that a recommended dietary allowance (RDA) of 15 μg/day for individuals aged 1–70 years will cover the requirement for 97.5 % of the population in the USA and Canada, corresponding to 25(OH)D concentrations of at least 50 nmol/L (Ross et al. 2011). Recently, the recommended intakes (RI) for vitamin D in the Nordic countries were increased from 7.5 to 10 μg/day for individuals aged 2–60 years. This will cover the requirement for 95 % of the Nordic population (Nordic Council of Ministers 2004; Nordic Council of Ministers 2014). Both IOM and Nordic nutrition recommendations (NNRs) 2012 based their RDA and RI on the relationship between 25(OH)D concentrations and bone health.

It is a public health concern that vitamin D intakes in most populations are lower than the RDA or RI (Andersen et al. 2005; Madsen et al. 2013; Nordic Council of Ministers 2014). Food fortification is an effective way to increase vitamin D intake in the general population (O’Mahony et al. 2011), thus ensuring that the general vitamin D intake aligns with the recommendations. During wintertime, a dietary intake of 10 μg/day is needed to maintain 25(OH)D concentrations around 50 nmol/L for the majority of the population in the Nordic countries. For people with little or no sun-exposure, an intake of 20 μg/day of vitamin D is recommended (Nordic Council of Ministers 2014). In Denmark, the mean dietary vitamin D intake is between 2.0 and 2.9 μg/day and does not meet the recommendations for the majority of the population (Tetens et al. 2011). Thus, during wintertime in Denmark, 50–90 % of the population will develop deficient vitamin D status between 30 and 50 nmol/L (Andersen et al. 2005; Thuesen et al. 2012; Madsen et al. 2013).

The main objective of this study was to assess the effect of real-life use of vitamin D_3_-fortified bread and milk on 25(OH)D concentrations in relation to common genetic variants in *CYP2R1* (rs10741657 and rs10766197) and *GC* (rs4588 and rs842999) in ethnic Danish families with dependent children during a 6-month winter period and furthermore to assess whether vitamin D supplementation will increase 25(OH)D concentration in those with genetically determined low 25(OH)D concentrations. A secondary objective was to evaluate the amount of vitamin D needed to maintain a sufficient 25(OH)D concentrations >50 nmol/L.

## Participants and methods

### Study design

The present study used data from the VitmaD intervention conducted in Gladsaxe Municipality in Denmark (latitude 56°N). The study design and methods are described in detail elsewhere (Madsen et al. 2013). Briefly, a double-blinded, randomized placebo-controlled intervention trial with apparently healthy ethnically Danish children and adults recruited as families was randomly allocated to either vitamin D_3_-fortified bread and milk or non-fortified placebo bread and milk during a 6-month winter period (September 2010 to April 2011) without sunlight exposure. The aim of the study design was to investigate a realistic D_3_-fortification strategy in real-life settings. Participants were instructed to replace their usual consumption of bread and milk with the products provided and in all other aspects, to live a normal life without changing any habits. The study was conducted according to the guideline in the Declaration of Helsinki, and the protocol was approved by the Danish ethics committee (H-4-2010-020) and registered in ClinicalTrials.gov (NCT01184716).

### Study population

A total of 201 Danish families with dependent children (*n* = 782), 4–60 years of age, randomly drawn from the Danish Civil Registration System, participated in the study. Inclusion criteria were age between 4 and 60 years and a permanent address in the Gladsaxe Municipality in Denmark. Exclusion criteria were pregnancy, disease or medication influencing vitamin D metabolism, including dietary supplements with >10 or >5 µg vitamin D/day for children or adults, respectively. All the adult participants and guardians of the children gave written informed consent.

### Vitamin D intakes

The participants’ vitamin D intakes were obtained from a self-administered web-based questionnaire based on a semi-quantitative food frequency questionnaire (Andersen et al. 2005) at baseline and at the end of the study. Dietary vitamin D intake was calculated based on the self-reported consumption frequencies and dietary contents of vitamin D (National Food Institute, Technical University of Denmark). Vitamin D intake from dietary supplements was calculated as self-reported frequency of use multiplied with the self-reported vitamin D content of the supplements. The contribution of vitamin D from intakes of vitamin D_3_-fortified bread and milk was calculated based on the self-reported consumption frequencies, amount and the measured vitamin D contents in the fortified products (5.2 ± 0.3 μg/100 g in wheat bread, 4.3 ± 0.3 μg/100 g in rye bread and 0.38 μg/100 mL in milk) (Madsen et al. 2013). The fortification strategy was to increase vitamin D intake to 7.5 μg/day as recommended in the Nordic nutrition recommendations (NNRs) until September 2013 (Nordic Council of Ministers 2004). Total vitamin D intake was estimated as the sum of dietary vitamin D, usage of multivitamin and vitamin D supplementation and furthermore intake of vitamin D_3_-fortified bread and milk for the fortification group.

### Biochemical analyses

Non-fasting venous blood samples were drawn, and serum and plasma were stored at −80 °C until analysis at Clinical Biochemical Department, Holbæk Hospital, Denmark. Measurements of serum 25(OH)D concentrations relied on the determination of both 25(OH)D_2_ and 25(OH)D_3_ and were conducted by isotope dilution liquid chromatography tandem mass spectrometry (LC–MS/MS). As primary calibrator, the standard reference material, vitamin D, in humans (SRM 972) from the National Institute of Standards and Technology was used. The analytic quality of 25(OH)D assay was assured by Vitamin D External Quality Assessment Scheme certification, and the mean bias was −3.2 %. The inter-assay CVs for 25(OH)D_2_ were 7.6 and 4.6 % at 43 and 150 nmol/L, respectively, and for 25(OH)D_3_ 2.2 and 2.8 % at 30 and 180 nmol/L, respectively, (Madsen et al. 2013). In Denmark, 25(OH)D concentrations <25 nmol/L are defined as vitamin D deficient, between 25 and 50 nmol/L as vitamin D insufficient and >50 nmol/L as vitamin D sufficient for the majority of the population (National Board of Health 2010). 25(OH)D concentrations can be divided by 2.496 to convert from nmol/L to ng/ml.

Plasma PTH levels (CV: 3.4 %) was measured by using immunology analyser Cobas e601 (Roche Diagnostics), and total calcium (CV 3.4 %) was measured by using a chemistry analyser Cobas c501 (Roche Diagnostics).

### SNP selection and genotyping

In a previous study (Nissen et al. 2014), we genotyped 25 SNPs in seven vitamin D-related genes (*CYP2R1*, *CYP24A1*, *CYP27B1*, *C10orf88*, *DHCR7/NADSYN1*, *GC* and *VDR*) selected based on the reports from two GWAS and several candidate gene studies. We found a strong association between common SNPs in *CYP2R1* and *GC* genes and baseline 25(OH)D concentrations in the presently studied 201 healthy Danish families with dependent children. We found that four SNPs, rs10741657 and rs10766197 in *CYP2R1* and rs4588 and rs842999 in *GC,* predicted baseline 25(OH)D concentrations. None of the four SNPs were in LD with each other: rs10741657 and rs10766196 (Pearson’s *r* = 0.60), rs10741567 and rs842999 (Pearson’s *r* = 0.03), rs10741657 and rs4588 (Pearson’s *r* = 0.10), rs10766197 and rs842999 (Pearson’s *r* = 0.09), rs10766197 and rs4588 (Pearson’s *r* = 0.05) and rs842999 and rs4588 (Pearson’s *r* = 0.0.31) were in LD. For the tri-allelic rs842999, there was a dose-dependent relationship between 25(OH)D concentrations and carriers of none, one or two copies of the G-allele and genotypes are presented as GG, GX and XX, where X represents C- or A-alleles.

DNA was purified from buffy coats as described by Miller et al. (1988). SNPs were genotyped using a Sequenom^®^ platform (San Diego, California) and the iPLEX Gold reaction. The SNPs and the primers used are listed in Supplementary Table 1. Each PCR reaction contained 10 ng genomic DNA, 0.5 U HotStart Taq (Qiagen), 1.25 × Enzyme Buffer (Qiagen), 3.5 mM MgCl_2_, 1 mM of each deoxynucleotide. The primers were added to a final concentration of 500 nM each. The PCRs were performed at the following cycling parameters: 15 min preheat to 94 °C, 45 cycles (20 s 94 °C, 30 s 56 °C, 1 min 72 °C) followed by 3 min 72 °C and stored at −20 °C. The PCR products were treated with shrimp alkaline phosphatase, dephosphorylate unincorporated dNTPs and extension with molecular weight-modified nucleotides were performed in concordance to the manufacturer’s recommendations. The PCRs were cleaned with resin and dispend on SpectroCHIP^®^ bioarrays. The SpectroCHIP^®^ bioarrays were placed in a MALDI-TOF mass spectrometer, and the results were analysed by MassARRAY Type 4.0 (Sequenom) (Nissen et al. 2014).

Of the 782 recruited children and adults, DNA was obtained from 769 participants (98.3 %). A total of 762 (99.1 %) were successfully genotyped. For quality control, 344 duplicated samples (44 %) were randomly placed throughout each of the 384-well plates and the reproducibility was 100 %. No deviation from Hardy–Weinberg equilibrium was observed for the adult population (*χ* ^2^ testing, *p* > 0.05).

### Statistical analysis

All statistical analyses were carried out using SAS Enterprise Guide 4.3 (SAS Institute, Inc., Cary. USA). Linear mixed models with family as a random factor were applied in all analyses to account for the non-independency of the participants. Before analysis, 25(OH)D concentrations and PTH levels were log-transformed to approximate a normal distribution and all means are presented as geometric means, unless otherwise specified. A nominal *p* value of 0.05 was considered statistically significant.

The following categorical variables were used: age (4–11, 12–17, 18–40, 41–60 years), sex (male, female), BMI (underweight, normal weight, overweight, obese) according to standards for children (Cole et al. 2000) and the WHO International standards for adults (World Health Organization 2000) measured at baseline, went on ski and sun vacation during the study period (yes, no), solarium use at least once a week (yes, no) and total calcium at baseline and at the end of the study. The continuous variables are log 25(OH)D concentrations and log PTH levels at baseline and at the end of the study, total vitamin D intake from diet, multivitamins and vitamin D supplements (μg/day).

A genetic risk score (GRS) was calculated as the sum of number of risk alleles. The GRS (range 0–8) was calculated as the sum of number of G-alleles of rs10741657, A-alleles of rs10766197, A-alleles of rs4588 and C/A-alleles of rs842999. A linear mixed model, adjusted for family and confounding variables, was fitted to the log 25(OH)D concentration with GRS as an explanatory factor. The adjusted mean concentration of 25(OH)D was calculated for each GRS. All the analyses were performed for control and fortification group and separately for adults and children.

Furthermore, each GRS category was stratified by quintile of total vitamin D intake (Q1: 0–2.9 μg/day; Q2: 3–7.4 μg/day; Q3: 7.5–9.9 μg/day; Q4: 10.0–14.9 μg/day; and Q5: >15.0 μg/day). Total vitamin D intake was estimated as the sum of dietary vitamin D, use of multivitamin and vitamin D supplementation and, for the fortification group, intake of vitamin D_3_-fortified bread and milk. The final concentration of 25(OH)D was estimated for each GRS by intake groups adjusted for family and confounding variables.

The prevalence (%) of participants with sufficient (>50 nmol/L) 25(OH)D concentrations was estimated for each GRS by intake groups adjusted for family and confounding variables.

## Results

Of the 782 recruited children and adults, 762 participants had complete questionnaire data, genotypes and 25(OH)D concentrations measured at baseline. At the end of the study, a total of 756 participants (control group *n* = 384 and fortification group *n* = 384) had complete questionnaire data, genotypes and 25(OH)D concentrations measured. Characteristics of the study population are listed in Table 1, as previously described in detail elsewhere (Madsen et al. 2013; Nissen et al. 2014). At baseline, participants in the control group had significantly higher total calcium levels (*p* = 0.0438) compared to participants in the fortification group, as previously reported (Madsen et al. 2013). Furthermore, there was a statistically significant difference between the control and fortification group for the use of solarium (*p* = 0.0059), and ski and sun vacation (*p* = 0.0006) during the intervention period as previously reported (Madsen et al. 2013).

**Table 1:** Basic characteristics of the study population (*n* = 762)

	Fortification group	Control group	*p* value
Participants (*n*)	377	385	–
Female/male (*n*)	191/186	199/186	0.7771
Age (*n*)			0.5430
4–10 years	94	91	0.5893
11–17 years	75	88	0.7064
18–40 years	111	87	0.4928
41–60 years	97	119	0.4802
BMI (kg/m^2^)	21.7 (21.17–22.3)	21.9 (21.3–22.4)	0.5515
25(OH)D (nmol/L)			
Baseline	72.7 (70.8–74.7)	71.1 (68.9–73.3)	0.4688
End	67.1 (65.2–69.0)	41.5 (39.6–43.5)	**<0.0001**
PTH (ng/L)			
Baseline	35.3 (34.1–36.5)	34.5 (33.3–35.7)	0.2473
End	36.8 (35.5–38.1)	40.1 (38.7–41.6)	**0.0199**
Total calcium (mmol/L)			
Baseline	2.44 (2.43–2.45)	2.45 (2.44–2.46)	**0.0438**
End	2.43 (2.42–2.44)	2.43 (2.42–2.44)	0.8165
Total vitamin D intake (μg/day)			
Baseline	2.9 (2.8–3.1)	2.7 (2.5–2.9)	0.4972
End	11.7 (11.0–12.4)	4.1 (3.8–4.5)	**<0.0001**
Supplement users (*n*)			
Baseline	127	127	0.7163
End	230	242	0.5991
Ski and sun vacation during the study (*n*)	135	100	**0.0006**
Solarium users during the study (*n*)	0	8	**0.0059**
Sunscreen use (*n*)			
Always/most times/sometimes/seldom	82/108/144/36	105/114/132/32	**0.3361**

All means are presented as geometric means with 95 % confidence interval in parentheses. Continuously variables are tested with *t* test, and categorical variable are tested with Chi-square

Bold numbers represent significant *P* values

In a previous study (Nissen et al. 2014), we found that at baseline, *CYP2R1* (rs10741657 and rs10766197) and *GC* (rs4588 and rs842999) were strongly associated with 25(OH)D concentrations among all participants (Table 2). At the end of the study, no associations between SNPs rs10741657 and rs10766197 in *CYP2R1* or rs4588 and rs842999 in *GC* and 25(OH)D concentrations were found for the control group. For the fortification group, rs10741657 in *CYP2R1* and rs4588 and rs842999 in *GC* were statistically significantly associated with 25(OH)D concentrations. The association with *CYP2R1* (rs10766197) was borderline significant (*p* = 0.0599). At the end of the study, total vitamin D intake (*p* < 0.0001) and 25(OH)D concentrations (*p* < 0.0001) were, as expected, significantly higher in the fortified group compared to the control group as previously reported (Madsen et al. 2013).

**Table 2:** The association between SNPs in *CYP2R1* and *GC* gene, and 25(OH)D concentrations at baseline (all) and at the end of the study (control/fortification)

Baseline						End of study				
Geometric mean 25(OH)D nmol/L (95 % CI)						Geometric mean 25(OH)D nmol/L (95 % CI)				
***CYP2R1***						**Group**				
rs10741657		**AA**	**GA**	**GG**	P_adj_		**AA**	**GA**	**GG**	P_adj_
	All	76.6 (73.0–80.5)	73.9 (71.8–76.1)	67.2 (65.0–69.5)	**<0.0001**	Control	40.6 (36.4–45.3)	43.1 (40.3–46.1)	39.8 (36.8–43.1)	0.1240
		(*n* = 125)	(*n* = 365)	(*n* = 268)			(*n* = 66)	(*n* = 175)	(*n* = 128)	
						Fortification	69.1 (64.3–74.3)	69.7 (67.0–72.5)	63.1 (60.4–66.0)	**0.0130**
							(*n* = 51)	(*n* = 171)	(*n* = 133)	
rs10766197		**GG**	**AG**	**AA**			**GG**	**AG**	**AA**	
	All	74.3 (71.6–77.1)	72.9 (70.8–75.1)	66.9 (64.2–69.8)	**<0.0001**	Control	41.5 (38.2–45.1)	42.6 (39.9–45.6)	38.7 (34.8–43.0)	0.1996
		(*n* = 221)	(*n* = 359)	(*n* = 177)			(*n* = 116)	(*n* = 181)	(*n* = 72)	
						Fortification	68.3 (64.6–72.1)	68.0 (65.3–70.8)	64.3 (61.0–67.7)	0.0599
							(*n* = 91)	(*n* = 164)	(*n* = 99)	
***GC***										
rs4588		**CC**	**CA**	**AA**			**CC**	**CA**	**AA**	
	All	75.1 (73.1–77.2)	69.9 (67.7–72.1)	61.2 (56.9–62.9)	**<0.0001**	Control	41.2 (38.7–44.0)	41.4 (38.5–44.5)	44.6 (37.1–53.5)	0.4163
		(*n* = 400)	(*n* = 303)	(*n* = 55)			(*n* = 193)	(*n* = 152)	(*n* = 24)	
						Fortification	70.9 (68.3–73.5)	64.6 (61.8–67.4)	55.0 (49.9–60.6)	**<0.0001**
							(*n* = 191)	(*n* = 137)	(*n* = 27)	
rs842999		**GG**	**GX (GA or GC)**	**XX (CC, CA, AA)**			**GG**	**GX (GA or GC)**	**XX (CC, CA, AA)**	
	All	75.4 (72.7–78.3)	72.7 (70.7–74.8)	65.0 (62.2–68.0)	**<0.0001**	Control	40.5 (37.1–44.2)	43.5 (40.8–46.5)	38.0 (34.4–42.0)	0.4099
		(*n* = 217)	(*n* = 383)	(*n* = 152)			(*n* = 104)	(*n* = 185)	(*n* = 79)	
						Fortification	72.4 (68.9–76.1)	66.8 (64.3–69.4)	60.2 (56.5–64.0)	**<0.0001**
							(*n* = 105)	(*n* = 179)	(*n* = 67)	

Bold numbers represent significant *P* values. Major, major homozygotes; het, heterozygotes; Minor, minor homozygotes

P_adj_ linear mixed models with family as a random factor, adjusted for age, sex, BMI, ski and sun vacation, total vitamin D intake estimated as the sum of dietary vitamin D, usage of multivitamin and vitamin D supplementation and for the fortification group intake of vitamin D_3_-fortified bread and milk

**Fig. 1:**
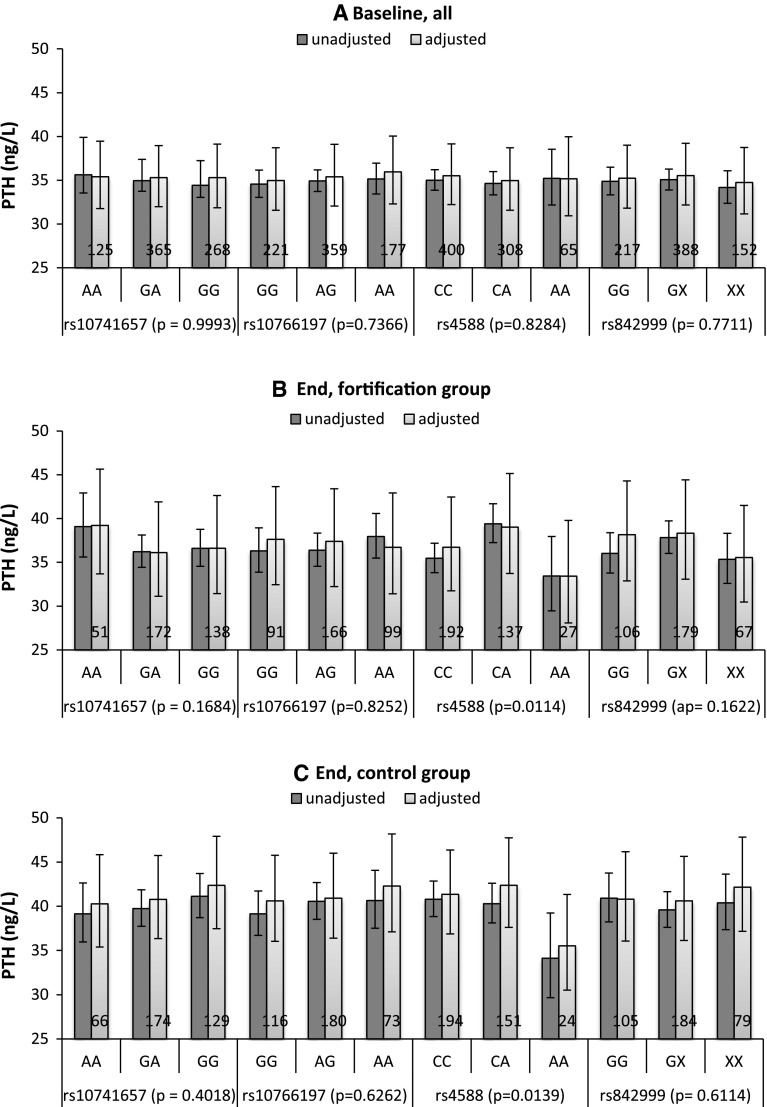
Association of rs10741657, rs10766197, rs4588 and rs842999 with PTH levels at baseline for all the participants and stratified by fortification and group control at the end of the study. Results are presented as unadjusted and adjusted geometric means. At baseline, the following variables were adjusted for age, sex, BMI, vacation and baseline total calcium, and at end of the study, the following variables were adjusted for age, sex, BMI, vacation, baseline 25(OH)D concentration, baseline PTH levels and end total calcium. Adjusted *p* values are given for each genotype. The *numbers* in the *columns* present the total numbers of participants carrying this genotype. *Error bars* indicate 95 % confidence interval. A statistically significant difference in PTH levels was observed for rs4588 in both the fortification and control group at the end of the study

There was no difference in PTH levels when stratified by rs10741657, rs10766197, rs4588 and rs842999 for all the participants at baseline (Fig. 1). As anticipated, PTH levels were significantly higher in the control group compared to the fortification group (*p* = 0.0199) at the end of the study (Table 1). Furthermore, there was a significant difference in PTH levels for rs4588 in both the fortification group (*p* = 0.0064) and control group (*p* = 0.0132) at the end of the study. Carriers of the rs4588-AA genotype had significantly lower PTH levels compared to carriers of either the rs4588-CA or rs4588-CC genotype.

**Fig. 2:**
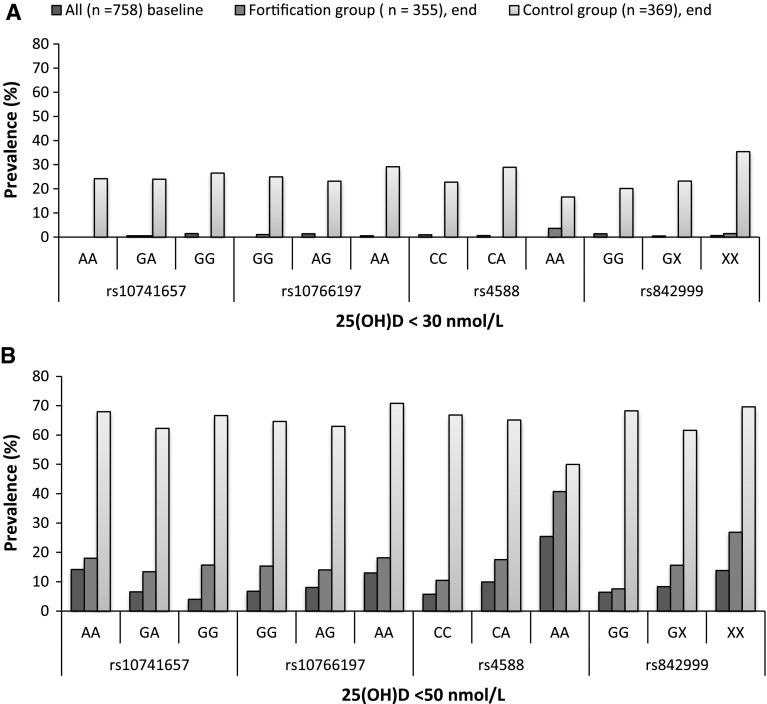
The prevalence (%) of <30 nmol/L **a** and <50 nmol/L **b** 25(OH)D concentrations in carriers of different genotypes of rs10741657, rs10766197, rs4588 and rs842999 at baseline for all the participants and at the end of the study stratified by control and fortification group. Cut-off value of 25(OH)D <50 nmol/L defines the requirement for optimal bone health for the majority of the population, and cut-off value <30 nmol/L defines the 25(OH)D concentration at which adverse effects on bone health may be expected (Ross et al. 2011)

The prevalence of participants with 25(OH)D concentration <30 nmol/L and <50 nmol/L was estimated for each genotype of rs10741657, rs10766197, rs4588 and rs842999 for all the participants at baseline and separately for the control and fortification group at the end of the study (Fig. 2a, b).

At baseline, there was no difference in the prevalence of participants having 25(OH)D concentrations <30 nmol/L stratifying by genotype rs10741657, rs10766197, rs4588 and rs842999 (*p* = 0.2269, 0.1715, 0.6953 and 0.5111), respectively. In contrast, there was significant difference in the prevalence of participants having 25(OH)D concentrations <50 nmol/L for rs10741657, rs4588 and rs842999 (*p* = 0.0004, <0.0001 and 0.0435), respectively, and rs10766197 was borderline significantly associated (*p* = 0.0743).

At the end of the study, for the fortification group, a significant difference in the prevalence of participants having 25(OH)D concentrations was found for rs4588 (*p* = 0.0023 < 30 nmol and for <50 nmol/L *p* = 0.0002) and rs842999 (*p* = 0.0029 < 50 nmol/L). No difference in prevalence was observed for rs10741657 and rs10766197 (*p* = 0.5830 and 0.2348 for <30 nmol/L and for <50 nmol/L *p* = 0.5466 and 0.6652), respectively. Furthermore, no difference in prevalence was found for rs842999 (*p* = 0.1194 for <30 nmol/L).

**Fig. 3:**
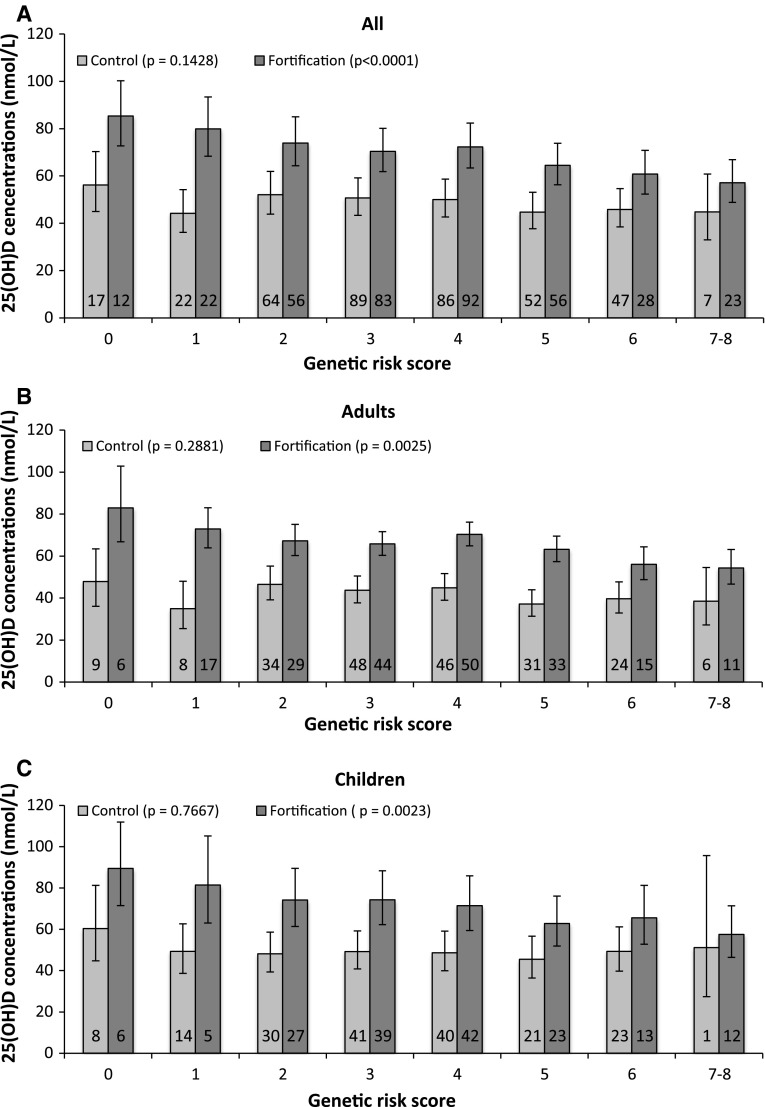
Estimated mean 25(OH)D concentrations at the end of the study for each genetic risk score category stratified by control and fortification group, separately for all (**a**), adults (**b**) and children (**c**). Individuals carrying 7 or 8 (7–8) risk alleles were combined due to small sample size. Genetic risk score (range 0 to 7–8) was calculated as the sum of number of G-alleles of rs10741657, A-alleles of rs10766197, A-alleles of rs4588 and C/A-alleles of rs842999. The *numbers* in the *columns* present the total numbers of participants carrying the risk score. *Error bars* indicate 95 % confidence interval

For the control group, only rs842999 <30 nmol/L was significant (*p* = 0.0455). No significant difference was observed for rs10741657, rs10766197 and rs4588 (*p* = 0.8694, 0.6130 and 0.2651 < 30 nmol/L and *p* = 0.5645, 0.4948 and 0.2641 for <50 nmol/L), respectively. Furthermore, rs842999 <50 nmol/L was also found to be non-significant (*p* = 0.3402). In general, the lowest prevalence of vitamin D deficiency <30 and <50 nmol/L was observed at baseline (*p* = 0.0001 and 0.0001), respectively. Participants in the control group presented more often with vitamin D deficiency <30 and <50 nmol/L compared to the fortification group (*p* = 0.0001 for <30 nmol/L and for <50 nmol/L *p* = 0.0001),

At the end of the study, to determine the combined contributions of rs10741657, rs10766197, rs4588 and rs842999, a GRS was calculated individually for the control and fortification group and separately for all, adults and children (Fig. 3a–c). Participants carrying seven or eight (7–8) risk alleles were combined due to small sample size. The coefficients for rs10741657, rs10766197, rs4588 and rs842999 were very similar in a mixed regression model including all SNPs, and therefore, it was not necessary to weight the different risk alleles by the correlation coefficient. A linear mixed model with family as a random factor, adjusted for age, sex, BMI, total vitamin D intake, and ski and sun vacation showed that for the control group, there was no difference in 25(OH)D concentrations for carriers of 0 to 7–8 risk alleles (*p* = 0.1428, 0.2881 and 0.7667) for all, adults and children, respectively. For the fortification group, there was a negative linear trend between 25(OH)D concentrations and carriers of 0 to 7–8 risk alleles for all, adults and children (*p* < 0.0001, 0.0025 and 0.0023, respectively). Overall, there was a mean difference in 25(OH)D concentrations of 28.2, 28.6 and 31.9 nmol/L between carriers of no risk alleles and carriers of all 7–8 risk alleles in all, adults and children, respectively. Overall, the same GRS pattern was observed for adults and children.

**Fig. 4:**
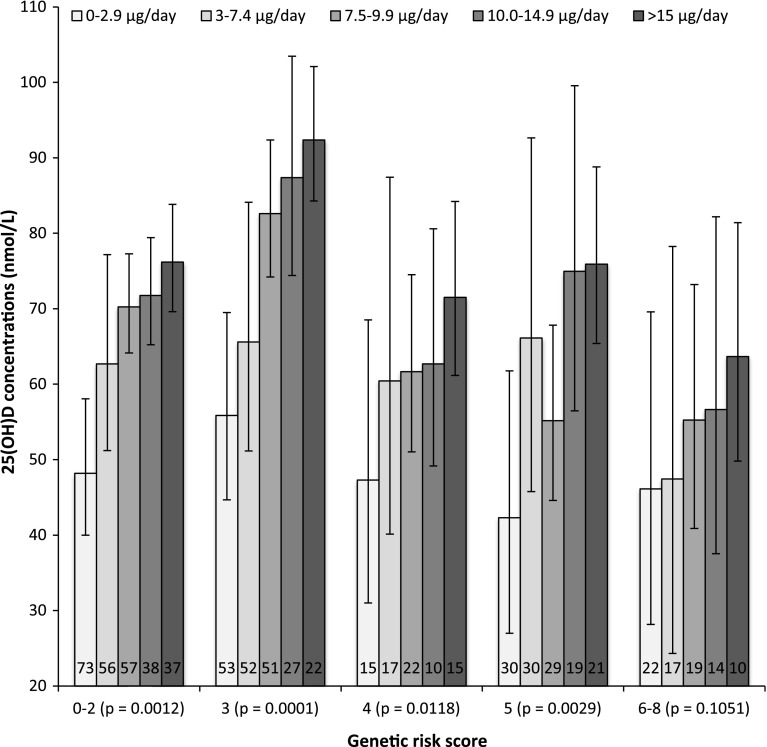
Mean 25(OH)D concentrations at the end of the study for each genetic risk score category stratified by total vitamin D intakes for the study population. Total vitamin D intake was estimated as the sum of dietary vitamin D, usage of multivitamin and vitamin D supplementation and, for the fortification group, intake of vitamin D_3_-fortified bread and milk. The following quintile stratification was used: quintile 1: 0–2.9 μg/day; quintile 2: 3–7.4 μg/day; quintile 3: 7.5–9.9 μg/day; quintile 4: 10.0–14.9 μg/day; and quintile 5: >15.0 μg/day. Genetic risk score (range 0–8) was calculated as the sum of number of G-alleles of rs10741657, A-alleles of rs10766197, A-alleles of rs4588 and C/A-alleles of rs842999. Individuals carrying 0, 1 or 2 (0–2) risk alleles and individuals carrying 6, 7 or 8 (6–8) risk alleles were combined due to small sample size after quintile stratification by total vitamin D intake. The *numbers* in the *columns* present the total numbers of participants carrying this risk score. *Error bars* indicate 95 % confidence interval

We estimated the effect of total vitamin D intake for each category GRS (range 0–8), for the combined contributions of rs10741657, rs10766197, rs4588 and rs842999 (Fig. 4). Each participant was stratified by quintile of total vitamin D intake. Total vitamin D intake was estimated as the sum of dietary vitamin D, use of multivitamin and vitamin D supplements and, for the fortification group, self-reported intake of vitamin D_3_-fortified bread and milk. Quintile stratification for total vitamin D intake was based on different RDA or RI: <3 μg/day (no supplementation), <7.5 μg/day (old NNRs 2004), <10 μg/day (present NNRs 2012), <15 μg/day (IOM) or >15 μg/day. The following quintile stratification cut-off values were used: quintile 1: 0–2.9 μg/day; quintile 2: 3–7.4 μg/day; quintile 3: 7.5–9.9 μg/day; quintile 4: 10.0–14.9 μg/day and quintile 5: >15.0 μg/day. The control and fortification groups were combined in the linear mixed model. Individuals carrying 0, 1 or 2 (0–2) risk alleles or individuals carrying 6, 7 or 8 (6–8) risk alleles were combined due to small sample sizes after quintile stratification by total vitamin D intake. A total of 25.1, 22.4, 23.4, 15.6 and 13.6 % of the adult participants carried 0–2, 3, 4, 5 or 6–8 risk alleles, respectively. The majority of the participants in the control group had low total vitamin D intake and were therefore primarily located in the first two quintiles. In general, there was a statistically significant, positive linear relationship between total vitamin D intake and 25(OH)D concentrations among carriers of 0–2, 3, 4 or 5 risk alleles, (*p* = 0.0012, 0.0001, 0.0118 and 0.0029, respectively). For individuals carrying 6–8 risk alleles, there was no statistically significant relationship between total vitamin D intake and 25(OH)D concentrations (*p* = 0.1051).

A the end of the winter season in Denmark, a total vitamin D intake of <3 μg/day was not sufficient for 95 % of the study population to achieve sufficient (>50 nmol/L) 25(OH)D concentrations, regardless of the number of risk alleles they carried (Fig. 4). For participants carrying 0–2 or 3 risk alleles, a total daily vitamin D intake between 3 and 7.4 μg seemed to be sufficient for 95 % of the study population to achieve sufficient 25(OH)D concentrations. For participants carrying four risk alleles, a total daily vitamin D intake >7.5 μg seemed to be sufficient for 95 % of the study population to achieve sufficient 25(OH)D concentrations. For participants carrying five risk alleles, a total daily vitamin D intake >10 μg seemed to be sufficient for 95 % of the study population to achieve sufficient 25(OH)D concentrations. For participants carrying 6–8 risk alleles, a total daily vitamin D intake >15 μg was almost enough for 95 % of the study population to achieve sufficient 25(OH)D concentrations.

**Fig. 5:**
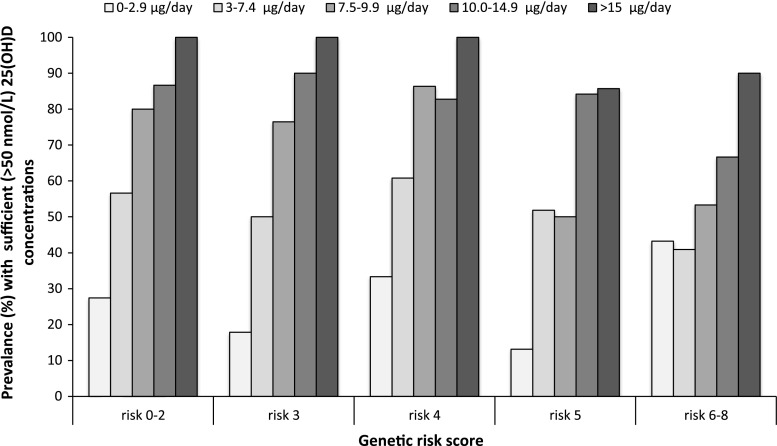
The prevalence (%) of sufficient 25(OH)D concentrations, defined as >50 nmol/L, for each genetic risk score category stratified by quintile of total vitamin D intake at the end of the study. Total vitamin D intake was estimated as the sum of dietary vitamin D, use of multivitamin and vitamin D supplements and, for the fortification group, intake of vitamin D_3_-fortified bread and milk. The following quintile stratification was used: quintile 1: 0–2.9 μg/day; quintile 2: 3–7.4 μg/day; quintile 3: 7.5–9.9 μg/day; quintile 4: 10.0–14.9 μg/day; and quintile 5: >15.0 μg/day. Genetic risk score (range 0–8) was calculated as the sum of number of G-alleles of rs10741657, A-alleles of rs10766197, A-alleles of rs4588 and C/A-alleles of rs842999. Individuals carrying 0, 1 or 2 (0–2) risk alleles and individuals carrying 6, 7 or 8 (6–8) risk alleles were combined due to small sample size after quintile stratification by total vitamin D intake

In addition, we determined the percentage of participants with sufficient 25(OH)D concentrations (Fig. 5). Sufficient 25(OH)D concentrations were achieved for all participants carrying 0–2, 3 or 4 risk alleles and who consumed >15 μg/day of vitamin D. For participants carrying 5 or 6–8 risk alleles, this fell to 86 and 90 %, respectively. Furthermore, sufficient 25(OH)D concentrations were achieved for 87, 90, 83, 84 and 67 % of the participants carrying 0–2, 3, 4, 5 or 6–8 risk alleles and who consumed 10–14.9 μg/day. This fell to 80, 76, 86, 50 and 53 % and 57, 50, 61, 52 and 41 % for participants carrying 0–2, 3, 4, 5 or 6–8 risk alleles and who consumed 7.5–9.9 μg/day or 3.0–7.4 μg/day of vitamin D, respectively.

## Discussion

In the present study, we show that genetic variation influences 25(OH)D concentrations considerably. Genetically predisposed individuals carrying 6–8 risk alleles of rs10741657 and rs10766197 in *CYP2R1* and rs4588 and rs842999 in *GC* need >15 μg/day or more vitamin D to reach 25(OH)D concentrations >50 nmol/L during winter. Furthermore, there was a statistically significant dose-dependent relationship between 25(OH)D concentration and total vitamin D intake for carriers of 0–5 risk alleles of SNPs rs10741657 and rs10766197 in *CYP2R1* and rs4588 and rs842999 in *GC*. A dose-dependent relationship was also observed for carriers of 6–8 risk alleles, but the increase in 25(OH)D concentrations was not statistically significant.

At baseline, our study showed that there was statistically significant difference in the prevalence of participants presenting with 25(OH)D concentration <50 nmol/L for rs10741657, rs10766197, rs4588 and rs842999. The significant differences in prevalence disappeared during the winter for the control group, but were maintained for rs4588 and rs842999 in the fortification group. For the fortification group, the highest prevalence of 25(OH)D <50 nmol/L was observed for the rs4588-AA genotype. In contrast, in the control group, rs4588-AA carriers had the lowest prevalence of 25(OH)D <50 nmol/L. This indicates that although carriers of the rs4588-AA genotype in the fortification group were more prone to be vitamin D deficient, rs4588-AA carriers in the control group were less prone to be vitamin D deficient. This may indicate that rs4588-AA carriers have a somewhat low but very stable 25(OH)D concentrations. Paradoxically, a recessive effect was observed for rs4588-AA carriers on PTH levels in both the fortification and control group at the end of the study. Participants with the rs4588-AA genotype have the lowest PTH levels and 25(OH)D concentrations compared to rs4588-CC or rs4588-CA carriers. Similar to our findings, Pekkinen et al. (2014) found a dose–response effect of rs4588 on PTH concentrations in 231 Finnish children and adolescents aged 7–19 years, with rs4588-AA carriers having the lowest PTH and 25(OH)D concentrations. Further studies are warranted to investigate the underlying biological mechanism of this observation.

At the end of the study, there was a pronounced positive effect of real-life usage of vitamin D_3_-fortified bread and milk on 25(OH)D concentrations. For the fortification group, 25(OH)D concentrations were significantly associated with rs10741657 in *CYP2R1*, and with rs4588 and rs842999 in *GC*. Furthermore, rs10766197 in *CYP2R1* was borderline significantly associated with 25(OH)D concentrations. These winter results resemble the results found at baseline (late summer) and indicate that when vitamin D is received primarily as vitamin D_3_-food fortification during the winter, the association between 25(OH)D concentrations and genetic variation observed at late summer for rs10741657 and rs10766197 in *CYP2R1* and rs4588 and rs842999 in *GC* is maintained. In contrast, the baseline association between 25(OH)D concentrations and rs10741657 and rs10766197 in *CYP2R1* and rs4588 and rs842999 in *GC* disappeared during the winter for the control group. Our findings are consistent with the findings from two previous studies (Gozdzik et al. 2011; Engelman et al. 2013). Gozdzik et al. (2011) found that rs4588 in *GC* was associated with 25(OH)D concentrations in Canadians of European descent during the fall (*p* = 0.009), but not during the winter (*p* = 0.535). Similarly, Engelman et al. (Engelman et al. 2013) found two SNPs in *GC* (rs4588 and rs7041) and four SNPs in *CYP2R1* (rs105000804, rs11023380, 2060763, 11023374) to be strongly associated with 25(OH)D concentrations in individuals whose blood was drawn in summer but not in individuals whose blood was drawn in winter month.

Engelman et al. (2013) performed a GRS for rs4588 in *GC* and rs2060793 in *CYP2R1*. The risk scores were highly significantly associated with 25(OH)D concentrations in individuals with high external source of vitamin D (>10 μg/day) but not in individuals with low external source of vitamin D (<10 μg/day). In addition, Gozdzik et al. (2011) found that vitamin D intake was significantly predictive of 25(OH)D concentrations in individuals carrying the rs4588 (T436 K) or in *GC* diplotypes during fall and winter. Our results support these findings by Engelman et al. (2013) and Gozdzik et al. (2011). We performed a GRS including the four SNPs rs10741657 and rs10766197 in *CYP2R1* and rs4588 and rs842999 in *GC*. For the fortification group, the GRS was highly significantly associated with 25(OH)D concentrations (*p* < 0.0001) but not for the control group (*p* = 0.1428) during winter. In general, children had higher mean 25(OH)D concentrations compared to adults. For the fortification group, an explanation could be that the children consumed more vitamin D_3_-fortified bread and milk compared to the adults. Approximately 90 % of the total intake of consumed bread and milk was the products provided by the study, with no difference in compliance between children and adults (Madsen et al. 2013). In general, the children were more often multivitamin users compared to the adults (Madsen et al. 2013).

When stratifying total vitamin D intake into quintiles, our data suggest that it is difficult to raise 25(OH)D concentrations to a sufficient level in participants carrying 6–8 risk alleles with vitamin D_3_-fortified bread and milk. A statistically non-significant increase in 25(OH)D concentrations was found comparing the lowest and highest quintile of vitamin D intake for participants carrying 6–8, but with a much lower rate (+Δ17.6 nmol/L) compared to participants carrying 0–2, 3, 4 or 5 risk alleles (+Δ28.8, 36.5, 24.2 and 33.6 nmol/L), respectively, (Fig. 4). Whether this also applies for vitamin D synthesized in the skin during UVB exposure remains to be further investigated. These increases are similar to the findings by Engelman et al. (2013). They found that among individuals carrying 3–4 risk alleles of *GC* (rs4588) and *CYP2R1* (rs2060793), the lowest increase in 25(OH)D concentrations was observed in individuals carrying 3–4 risk alleles (+Δ16.7 nmol/L) compared to individuals with fewer risk alleles (+Δ27.7 nmol/L).

In our study population, 67 % of the participants carrying 6–8 risk alleles had sufficient 25(OH)D concentrations in contrast to 87, 90, 83 and 84 % for participants carrying 0–2, 3, 4 or 5 risk alleles, respectively, when following IOMs RDA of 15 μg/day for individuals aged 1–70 years. Following the Nordic countries RI of 10 μg/day for individual aged 2–60 years, only 50 and 53 % of the participants carrying 5 or 6–8 risk alleles, respectively, had sufficient 25(OH)D concentrations compared to 80, 76 and 86 % of the participants carrying 0–2, 3 or 4 risk alleles, respectively. This indicates that genetic predisposition may have a large impact on 25(OH)D concentrations. Participants having a high GRS may need a higher amount of vitamin D supplementation than participants carrying a lower GRS in order to reach sufficient 25(OH)D concentrations. We provide evidence that participants with different genetic profiles need different amounts of vitamin D supplementation to achieve sufficient 25(OH)D concentrations. Epidemiological studies have found association between blood levels of vitamin D concentrations and risk of cancer, but the significance of genetically determined low vitamin D concentration is not clear.

In agreement with our findings, Cranney et al. (2007) concluded that vitamin D_3_-doses of 10–20 μg/day may be insufficient to prevent vitamin D deficiency in at-risk individuals. Cashman et al. (2011) concluded that for a population to achieve 25(OH)D concentrations of 50 nmol/L, an average intake of 9 μg/day vitamin D was needed. Nevertheless, taking inter-individual variation into account 23.5 μg/day of vitamin D_3_ was needed for 95 % of the population to reach a 25(OH)D concentration of 50 nmol/L. Engelman et al. (2013) found that all of the individuals with no risk alleles of rs4588 and rs2060793 who consumed at least 17 μg/day (670 IU/day) had 25(OH)D >50 nmol/L. This fell to 84, 72 and 62 %, respectively, for individuals carrying 1, 2 or 3–4 risk alleles who also consumed at least 17 μg/day.

Our study has several strengths in that we ensured a large age span (4–60 years), had both genders represented, and both children and adults were included due to the family-based design (Madsen et al. 2013). 25(OH)D concentrations were measured by a specific analytical method (LC–MS/MS). We took into account that non-genetic factors such as vitamin D intake and season are known to influence 25(OH)D concentrations. We estimated total vitamin D intake, and blood samples were drawn during the same seasons for all the participants. A disadvantage is that some of the known predictors of 25(OH)D concentration were quantified by self-reported questionnaire data.

In summary, we found that after consuming vitamin D_3_-fortified bread and milk during a winter season, the effect of genetic variation in the *CYP2R1* and *GC* genes on 25(OH)D concentrations resembles the results found in late summer. The association with genetic variation observed for *CYP2R1* and *GC* genes in late summer disappeared during the winter season for the control group. We found that carriers of the rs4588-AA genotype had the highest prevalence of 25(OH)D concentration <50 nmol/L at baseline and at the end of the study for the fortification group. In contrast, rs4588-AA carriers in the control group had the lowest prevalence. It seems like rs4588-AA carriers have a low but very stable 25(OH)D concentration, and interestingly, also low PTH level.

In this study, we demonstrated that carriers of a high GRS of *CYP2R1* (rs10741657 and rs10766197) and *GC* (rs4588 and rs842999) are more prone to be vitamin D deficient compared to carriers of a low GRS. Furthermore, carriers of a high GRS may need a higher amount of vitamin D_3_ supplementation to achieve sufficient 25(OH)D concentrations. Importantly, for public health recommendations, it seems that with increasing vitamin D intake, genetically determined low risk carriers with sufficient 25(OH)D concentrations achieve even higher 25(OH)D concentrations with the used real-life vitamin D_3_-fortification model.

## Electronic supplementary material

Below is the link to the electronic supplementary material.

Supplementary material 1 (DOCX 41 kb)

## Abbreviations

DBP: Vitamin D-binding protein
GC: Vitamin D-binding protein gene or group-specific component
GRS: Genetic risk score
GWAS: Genome-wide association studies
IOM: Institute of Medicine
LC–MS/MS: Isotope dilution liquid chromatography tandem mass spectrometry
LD: Linkage disequilibrium
NNRs: Nordic nutrition recommendations
PTH: Parathyroid hormone
RDA: Recommended dietary allowance
RI: Recommended intakes
25(OH)D: 25-Hydroxyvitamin D
SNPs: Single-nucleotide polymorphisms
UVB: Ultraviolet B radiation
