# Supplementary material for: Real-life use of vitamin D3-fortified bread and milk during a winter season: the effects of CYP2R1 and GC genes on 25-hydroxyvitamin D concentrations in Danish families, the VitmaD study
Source: Genes Nutr. 2014 Jun 17;9(4):413. doi: 10.1007/s12263-014-0413-7 (PMC4169060; doi:10.1007/s12263-014-0413-7)
Supplement: Supplementary file 1 — Supplementary material 1 (DOCX 41 kb) [file 12263_2014_413_MOESM1_ESM.docx]

**Supplementary Table 1:** *GC* and *CYP2R1* primers

| **SNP** | **iPlex primer 1** | **iPlex primer 2** | **Extension primer** |
| --- | --- | --- | --- |
| rs4588 | ACGTTGGATGTTTTTCAGACTGGCAGAGCG | ACGTTGGATGCTTGTTAACCAGCTTTGCC | GAAAGCTTTGCCAGTTCC |
| rs842999 | ACGTTGGATGTGAGAATATTAAGCACCGAG | ACGTTGGATGCTAGTCTTACATATATCAG | CTAGTCTTACATATATCAGAAATTG |
| rs10741657 | ACGTTGGATGGGTGGTTGGGGAGATACTTT | ACGTTGGATGCAGCTCCAATGTCATCTTCC | TTCCTTGACAGCCCT |
| rs10766197 | ACGTTGGATGAGCTTGGTCCTTTCTGTATC | ACGTTGGATGGTACAATTTGGAACACTCCAG | ACGCCAGTTAATTAGAGATCTTTAAACT |
